# Social determinants of health and outcome of extremely preterm infants: A Swiss population-based study

**DOI:** 10.1371/journal.pone.0344616

**Published:** 2026-04-21

**Authors:** Marion Decaillet, Timea Hasenauer, Juliane Schneider, Myriam Bickle Graz

**Affiliations:** 1 Clinic of Neonatology, Department of Mother-Woman-Child, Lausanne University Hospital and University of Lausanne, Lausanne, Switzerland; 2 Department of Radiology, Lausanne University Hospital and University of Lausanne, Lausanne, Switzerland; 3 The Sense Innovation and Research Center, Lausanne and Sion, Switzerland; 4 Faculty of Biology and Medicine, Lausanne University Hospital, Switzerland; Monroe Carell Junior Children#39;s Hospital at Vanderbilt, UNITED STATES OF AMERICA

## Abstract

**Background and aims:**

Social determinants of health (SDoH) are associated with the outcome of preterm infants, but data in Switzerland are lacking. The aim of this study was to assess the associations of maternal nationality, parental socioeconomic status (SES) and language, with perinatal and 18-month-old outcomes of extremely preterm infants in Switzerland.

**Methods:**

Surviving extremely preterm infants born in our tertiary care neonatal centre between 2009–2020 were evaluated at the age of 18 corrected months using Bayley Scales of Infant Development, and neurological examination. We analyzed differences in outcomes according to maternal nationality, socioeconomic status and language.

**Results:**

In the neonatal period, among 408 (46% female) patients (median 26 6/7 weeks (25 4/7–27 2/7)), median birthweight 790g (669-942g), nationality was associated with the rate of multiplets, (34% in Swiss vs 27% for European, 14% extra-European, *p* = .012). Parental SES was associated with the rate of multiplets, (45%, mid SES 28%, low SES 19%, *p* < 0.001), with having oxygen at 36 weeks (high SES 32%, mid SES 56%, low SES 19%, *p* = .01), and with the rate of feeding with mother’s own milk (high SES 80%, mid SES 67%, low SES 42%, *p* < .001). Parental language was not associated with neonatal outcomes. At 18 months, the rate of neurodevelopmental impairment was similar across the 3 SDoH, but the median cognitive score was significantly higher in Swiss than in extra-European patients (100(95–115) vs 90(85–100), *p* = .002), and in middle SES compared to low SES patients (100(95–110) vs 95(85–100), *p* = .031). The median cognitive (100(90–111) vs 95(85–100), *p* = .009) and language scores (91(83–103) vs 86(77–94), *p* = .002) of children of French speaking families were higher than non-French speaking families.

**Conclusion:**

Maternal nationality, parental SES and parental language were minimally associated with perinatal outcomes of EPT infants. However, these 3 SDoH were significantly associated with neurodevelopmental outcomes at 18 months.

## Introduction

The last 10 years have seen the emergence, mostly from the United States of America, of a body of work examining the additional influence of social determinants of health (SdOH) on the management and outcome of preterm infants [1], which has been extensively studied [2,3]. Factors influencing the short and long-term outcome of preterm infants include antenatal steroids, mode [4] and place of delivery [5], gestational age (GA), birth weight, sex, and medical complications such as sepsis, necrotizing enterocolitis, brain injury, retinopathy of prematurity (ROP), provision of mother’s own milk (MOM) [6] and lung disease [7,8]. These factors are associated with the risk of adverse outcome, including neurodevelopmental impairment (NDI). Socioeconomic and ethnic disparities have been consistently linked to health outcomes across several studies. Research in the United States shows that ethnicity and neighborhood are associated with mortality and overall outcome [9–11]. In the UK, socio-economic and ethnic disparities correlate with a higher rate of preterm birth and poorer neurodevelopment [12,13]. Also in the UK, Beauregard showed a synergetic effect of preterm birth and poverty on cognitive development [14] until school age. In Italy [15], migrant status has been related to perinatal health and neurodevelopmental outcome of preterm infants, the authors highlight how the interplay of the country of origin with the feeding regimen (mother’s own milk (MOM) vs. formula) are associated with neurodevelopment. In Switzerland, recent national population-based studies have demonstrated the association of both national origin and socioeconomic status (SES) with perinatal outcomes, including preterm birth, low birthweight and mortality [16,17].

In sum, SDoH have been linked to the management and outcomes of preterm infants across various contexts, and identified as a significant factor influencing the incidence of short- and long-term morbidity [12], which may lead to long-term NDI and disabilities. The association of SDoH with these long-term outcomes has seldom been reported in Europe and has not been studied in Switzerland. The aim of the present study was thus to examine the specific associations of these SDoH (i.e., maternal nationality, parental SES and language), with neonatal management, and short- and long-term outcome of extremely preterm (EPT) infants (i.e., born before 28 weeks of gestation) in a level III neonatal center.

## Materials and methods

The Swiss Society of Neonatology maintains a national register and with informed parental consent, the different centers prospectively collect perinatal data of infants liveborn in one of the 9 neonatal intensive care centers with a gestational age < 32 0/7 weeks, and neurodevelopmental follow-up data for infants < 28 weeks. Neonatal and follow-up data are securely recorded in the national database for quality control [18,19] and research purpose [20]. This study was set in one tertiary neonatal intensive care unit in the French-speaking part of Switzerland, caring for a population including all patients from the cantons of Vaud, and a proportion of patients from cantons Valais, and Fribourg.

### Data collection

Population: All neonates born before 28 completed weeks of gestation between January 2009 and December 2020, admitted to our tertiary neonatal care unit were eligible for this study. Patients were not included if parents had denied consent. Informed consent was oral before 2015 and written after 2015 (ethics approval KEK-ZH-Nr 2014–0552 and KEK-ZH-Nr 2014–0551). Ethical approval was granted for the access and use of data also without consent, so as to include deceased patients without having to contact parents. With the same aim, authors could access the hospital administrative database to complete the nationality database, and could thus identify individual participants during data collection. The database was accessed on the 12.12.2023, after final ethics approval.

During the study period, infants born between 22 0/7 and 23 6/7 weeks were not included in the study population, as care management was generally limited to palliative care following guidelines from the Swiss Neonatal Society [21].

Neonatal data were mostly extracted from the national register, and missing data were retrieved from the regional hospital database. Neonatal variables included death, GA, birth weight and birthweight z-score, gender, mode of delivery (i.e., cesarean section or vaginal), single or multiple pregnancies, place of birth, administration of prenatal steroids, bronchopulmonary dysplasia defined as supplemental oxygen requirement at 36 weeks of post-menstrual age (PMA), treated ROP, treated necrotizing enterocolitis, confirmed neonatal sepsis, abnormal head ultrasound (HUS) (i.e., cystic leukomalacia or severe brain hemorrhage grade > 2 according to Papile [22])), feeding with MOM at discharge from primary hospital, and discharge destination (i.e., home or transfer to another hospital). The information about maternal nationality, family SES and language were usually collected at follow-up. SES was defined according to the Largo score [23], which involves the mother’s educational level (from 1 to 6) and the professional status of the father (from 1 to 6), 2 being the highest SES and 12 the lowest. We categorized the Largo score into 3 groups: 2–5 (high), 6–8 (middle) and 9–12 (low). There were 59 different maternal nationalities which we grouped into Swiss, European, and non-European for comparison with the perinatal outcome data of Wanner [16]. Regarding language, parents reported 35 different languages, which we grouped into French (i.e., the local language) if at least one of the parents spoke it, and Others (i.e., all the other languages).

At the age of 18–24 corrected months, children were assessed with a detailed neurological examination, including assessment of hearing and vision, and their neurodevelopment was assessed with the Bayley Scales of Infant Development, 2^nd^ (36% of children) or 3^rd^ (64%) edition (BSID-II and BSID-III, respectively)[24–26]. If the child presented with cerebral palsy (CP), it was graded according to Palisano [27]. The neurodevelopmental outcomes assessed for comparison were the results of the BSID-II or III [19]), and a composite outcome of NDI, defined by either BSID-II mental index score <70, or a BSID-III cognitive score <80, or a BSID-III motor composite score <78 [26], or a BSID-III language composite score <85, or CP grade >1, or visual impairment with perception of light only or blindness, or absence of hearing despite hearing aids.

### Statistical analysis

Descriptive statistics were used to summarize the neonatal and follow-up data. Continuous variables are presented as means and standard deviations when normally distributed, or as medians and interquartile ranges (i.e., 1^st^ quartile – 3^rd^ quartile) if they are not. Categorical variables are described using proportions.

We analyzed differences in short- and long-term outcomes according to the variables of interest (i.e., maternal nationality, parental SES and language). Regarding maternal nationality and parental SES, differences between categorical variables were assessed with Chi-squared tests, and between continuous variables with one-way ANOVA when normally distributed, or Kruskal-Wallis tests otherwise. For significant differences, Dwass-Steel-Critchlow-Fligner pairwise comparisons were performed as post-hoc tests. For parental language, Chi-squared tests and t-tests were computed. Additionally, the false discovery rate was computed to account for multiple comparisons.

In addition, given that multiple pregnancies may represent a significant risk factor for neonatal mortality [28,29] and that its incidence varied depending on maternal nationality and parental SES, a generalized linear model adjusted for the presence of multiplets was computed for this outcome.

The statistical analyses were done with Jamovi (version 2.4) [30] and R (version 4.4.0) [31]. We followed the STROBE (Strengthening the reporting of observation studies in epidemiology) guidelines [32].

## Results

**Fig 1:**
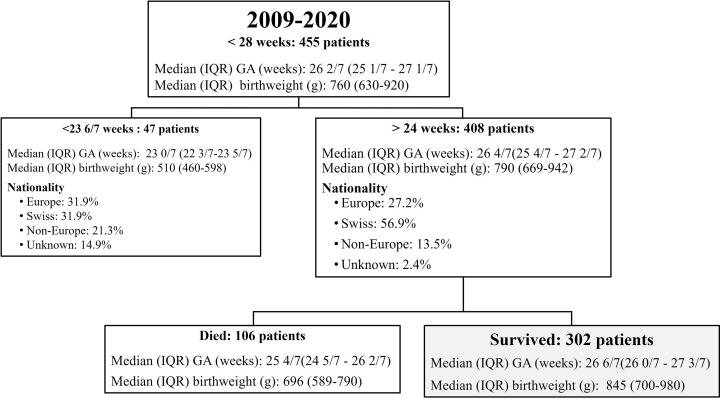
Flowchart of extremely preterm infants born between January 2009 and December 2020 in our tertiary neonatal care unit and the subsequent studied population.

Among 455 patients born before 28 weeks of gestation during the study period, 47 (10.3%) were born before 23 6/7 weeks of gestation and offered palliative care (2.13% of survival). Among the 408 patients born between 24 0/7 and 27 6/7 weeks of gestation, the survival rate was 74.02% (for details, see **Fig 1**).

### Neonatal characteristics

The medical and demographic characteristics are described in **Table 1**, **Table 2**, and **Table 3**, according to maternal nationality, parental SES and language, respectively. Overall, the neonatal characteristics were found to be comparable across the different nationalities, SES, and languages, except for the rate of multiple pregnancies, the administration of oxygen at 36 weeks PMA, and the number of patients receiving MOM at discharge. The odds of multiple pregnancies were higher in Swiss patients compared to European and, and non-European patients, and higher in high SES than middle SES patients, and low SES patients. The odds of having oxygen at 36 weeks PMA for patients with middle SES were higher than for patients with high and low SES. The probability of feeding with MOM was higher for infants of high SES and middle SES compared to low SES. Finally, parental language was not associated with any short-term outcome.

**Table 1:** Neonatal characteristics and morbidities according to maternal nationality.

	All > 24 weeks PMA n = 408	Swiss n = 235	Europe n = 113	Non-European n = 56	χ^2^ or F	*p*-value	*p*-value corrected	Cramér’s V or ε^2^
**Inborn, n(%)**	384(94)	219(93)	108(96)	54(98)	1.35	.614	.809	0.06
**Death, n(%)**	106(26)	55(23)	30(27)	18(32)	1.91	.377	.809	0.07
**SES total score, median(IQR)**	6(4-8)	5(4-7)	8(6-10)	8(6-10)	52.7	<.001***	<.001***	0.158
**Language**								
**French**	230 (67)	176 (88)	25 (27)	29 (62)	107	<.001***	<.001***	0.561
**Others**	111 (33)	25 (12)	68 (73)	18 (38)				
**Prenatal steroids, n(%)**	334(82)	197(84)	92(81)	43(77)	1.59	.451	.809	0.06
**C-section, n(%)**	278(68)	164(70)	74(66)	37(66)	0.77	.681	.809	0.04
**Multiplets, n(%)**	118(29)	79(34)	31(27)	8(14)	8.41	.012*	.076	0.14
**Birth weight (grams), median(IQR)**	790(669-942)	800(690-949))	750(630-930)	836(646-962)	2.26	.322	.809	0.006
**Gestational age, weeks, median (IQR)**	26 6/7(25 4/7 – 27 2/7)	26 4/7(25 5/7– 27 2/7)	26 3/7(25 3/7– 27 2/7)	26 2/7(25 4/7– 27 2/7)	1.39	.498	.809	0.003
**Sex, female, n(%)**	186(46)	103(44)	55(49)	28(50)	1.24	.543	.809	0.06
**Proven sepsis, n(%)**	110(38)	63(37)	36(47)	10(26)	5.28	.076	.353	0.135
**Necrotizing enterocolitis, n(%)**	57(14)	32(15)	15(14)	9(18)	0.49	.749	.837	0.04
**Cystic leukomalacia, n(%)**	10(3)	7(3)	2(2)	1(2)	0.607	.898	.932	0.040
**Severe Hemorrhage, n(%)**	68(18)	35(16)	20(19)	12(23)	1.61	.415	.809	0.065
**Abnormal head US, n(%)**	186(46)	108(49)	51(47)	25(50)	0.142	.932	.932	0.02
**Treated ROP, n(%)**	18 (5)	9 (4)	7 (7)	2 (4)	1.08	.609	.809	0.054
**Oxygen at 36 weeks, n(%)**	111(29)	71(32)	26(24)	13(26)	2.63	.284	.809	0.083
**Discharged, n(%):**								
**- home**	141(35)	76(32)	40(35)	24(43)	7.61	.093	.353	0.10
**- other hospital**	162(40)	105(45)	43(38)	14(25)				
**Mother’s own milk at discharge, n(%)**	208(67)	127(70)	55(65)	25(66)	0.77	.673	.809	0.05

*Note.* The column for *All > 24 weeks PMA* represents all EPT patients born at more than 24 weeks, regardless of maternal nationality, parental SES or language. *p < .05, **p < .01, ***p < .001.

**Table 2:** Neonatal characteristics and morbidities according to parental Socioeconomic status (SES).

	High SES n = 128	Middle SES n = 134	Low SES n = 72	χ^2^ or F	*p*-value	*p*-value corrected	Cramér’s V or ε^2^
**Inborn, n(%)**	122(95)	126(94)	69(96)	0.39	.854	.887	0.03
**Death, n(%)**	16(13)	13(9.7)	14(19)	3.99	.134	.318	0.109
**Nationality**							
**Swiss**	101 (79)	78 (58)	17 (24)	59.4	<.001***	<.001***	0.298
**Europe**	20 (16)	39 (29)	35 (48)				
**Other countries**	7 (5)	17 (13)	20 (28)				
**Language**							
**French**	98 (82)	85 (66)	28 (42)	30.9	<.001***	<.001***	0.313
**Other**	22 (18)	44 (34)	39 (58)				
**Prenatal steroids, n(%)**	112(88)	123(92)	58(81)	5.50	.066	.209	0.13
**C-section, n(%)**	98(77)	92(69)	49(68)	2.56	.282	.412	0.09
**Multiplets, n(%)**	57(45)	38(28)	14(19)	15.1	<.001***	0.003**	0.21
**Birth weight (grams), median(IQR)**	848(720-971)	783(650-942)	855(700-954)	4.12	.128	.318	0.01
**Gestational age, weeks, median(IQR)**	26 5/7(26 0/7-27 3/7)	26 4/7(25 3/7 - 27 2/7)	27 0/7(26 0/7 – 27 2/7)	0.21	.214	.377	0.01
**Sex, female, n(%)**	58(45)	66(49.3)	37(52)	0.92	.644	.779	0.05
**Proven sepsis, n(%)**	36(38)	41(40)	18(36)	0.217	.887	.887	0.03
**Necrotizing enterocolitis, n(%)**	15(12)	22(17)	10(15)	1.03	.608	.779	0.06
**Cystic leukomalacia, n(%)**	4(3)	3(2)	3(4)	0.656	.656	.779	0.045
**Severe Hemorrhage, n(%)**	15(2)	18(14)	11(16)	0.504	.762	.852	0.039
**Abnormal head US, n(%)**	51(41)	61(46)	38(55)	3.47	.176	.372	0.10
**Treated ROP, n(%)**	9 (7)	8 (6)	1 (2)	2.88	.237	.377	0.095
**Oxygen at 36 weeks, n(%)**	32(26)	56(42)	19(28)	8.84	.013*	.049*	0.165
**Discharged, n(%)**							
**home**	49(38)	58(43)	31(43)	5.72	.238	.377	0.09
**other hospital**	64(50)	63(47)	27(38)				
**Mother’s own milk at discharge, n(%)**	90(80)	81(67)	25(42)	26.4	<.001***	<.001***	0.300

Note. *p < .05, **p < .01, ***p < .001.

**Table 3:** Neonatal characteristics and morbidities according to parental language.

	Language = French N = 230	Language = other N = 111	χ^2^ or t	*p*-value	*p*-value corrected	Cramér’s V or Cohen’s d
**Inborn, n(%)**	217(94)	106(96)	0.197	.799	.910	0.02
**Death, n(%)**	34(15)	13(12)	0.594	.505	.910	0.04
**Nationality**						
**Swiss**	25 (23)	176 (76)	107	<.001***	<.001***	0.561
**Europe**	68 (61)	25 (11)				
**Other countries**	18 (16)	29 (13)				
**SES total score, median(IQR)**	6 (4-7)	8 (6-10)	5.29	<.001***	<.001***	0.642
**Prenatal steroids, n(%)**	201(87)	98(88)	0.06	.862	.910	0.02
**C-section, n(%)**	168(73)	78(70)	0.29	.608	.910	0.03
**Multiplets, n(%)**	67(29)	43(39)	3.16	.084	.399	0.10
**Birth weight (grams), median(IQR)**	800 (690-938)	840(668-1015)	0.45	.502	.910	0.001
**Gestational age, weeks, median(IQR)**	26 5/7 (25 6/7 – 27 2/7)	26 5/7 (25 6/7 – 27 3/7)	0.37	.831	.910	0.001
**Sex, female, n(%)**	101(44)	54(49)	0.62	.486	.910	0.04
**Proven sepsis, n(%)**	79(43)	23(31)	3.33	.068	.399	0.113
**Necrotizing enterocolitis, n(%)**	36(16)	12(11)	1.49	.248	.673	0.07
**Cystic leukomalacia, n(%)**	6(3)	4(4)	0.261	.733	.910	0.028
**Severe Hemorrhage, n (%)**	33(15)	18(16)	0.207	.631	.910	0.025
**Abnormal head US, n(%)**	111(49)	44(40)	2.36	.131	.498	0.08
**Treated ROP, n(%)**	12 (5)	6 (6)	0.02	1	1	0.008
**Oxygen at 36 weeks, n(%)**	74(33)	34(31)	0.097	.804	.910	0.017
**Discharged, n(%)**						
**home**	91 (40)	46(41)	0.46	.819	.910	0.04
**other hospital**	106(46)	52(47)				
**Mother’s own milk at discharge, n(%)**	138 (70)	60(62)	2.01	.236	.673	0.075

Note. *p < .05, **p < .01, ***p < .001.

Additionally, there were significant associations within the three SDoH.

Swiss patients had a higher SES compared to European and non-European patients. The odds of speaking French were higher for high SES patients compared to middle SES and low SES patients. Similarly, the odds were higher for middle SES compared to low SES patients. In addition, Swiss patients were more likely to speak French than European and non-European patients. However non-European patients were found to be more likely to speak French than European patients. Adjustment for multiple pregnancies did not change the results for the outcome of “death”. In addition, when corrected for multiple comparisons, the results remained similar, but for the multiplet rate which became comparable across nationalities.

### 18-month assessment

Infants were examined at a mean age of 19.5 months (1.9 months) of corrected age. The follow-up rate among survivors was 92.4%. Among them 24% of infants were diagnosed with a composite outcome of NDI (i.e., visual impairment, hearing loss, CP grade >1, or developmental delay) and 46% received at least one neurodevelopmental therapy. These rates were not different across the different nationalities, SES or languages.

**Fig 2:**
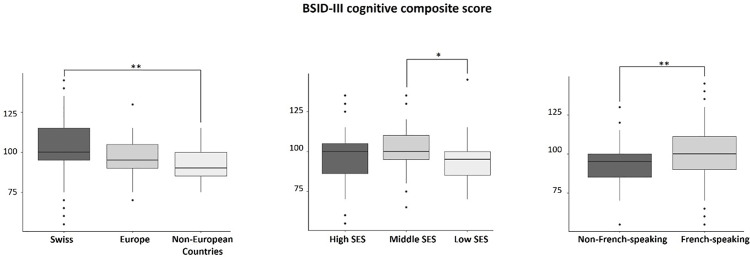
Median of BSID-III cognitive scores as a function of maternal nationality, parental SES and language. Note. *p <.05, **p < .01.

**Fig 3:**
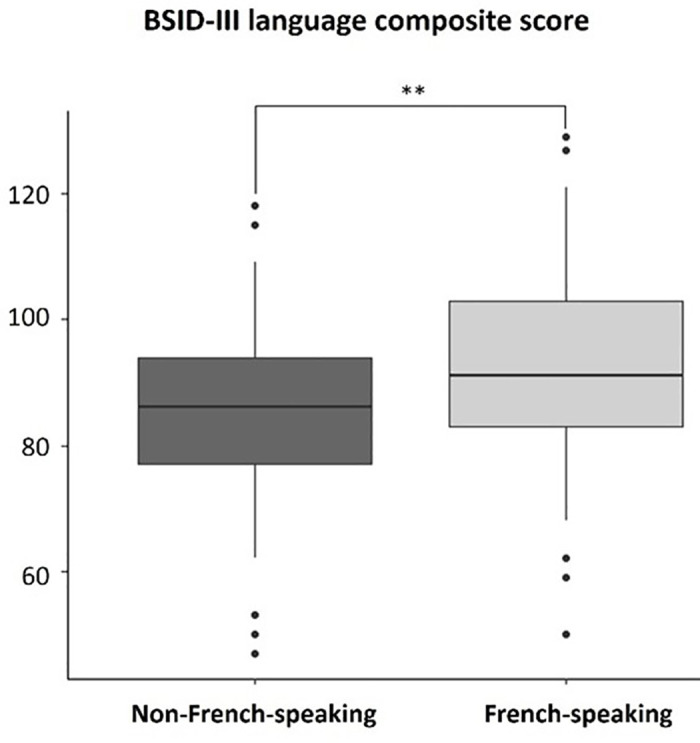
Median of BSID-III language composite scores as a function of parental language.

Neurodevelopmental characteristics are described in **Table 4**, **Table 5**, and **Table 6**, according to the different nationalities, SES, and languages. The median BSID-III cognitive score was significantly higher for Swiss patients compared to non-European patients, and for patients with middle SES compared to patients with low SES. In addition, infants from French-speaking families had significantly higher median scores compared to non-French-speaking families on the BSID-III cognitive score, and on the BSID-III language composite score (see **Fig 2** and **Fig 3**). Other neurodevelopmental outcomes were similar between groups. Results were comparable when data were corrected for multiple comparisons, except for the BSID-III cognitive score, which no longer differed according to the SES.

**Table 4:** Neurodevelopment according to maternal nationality.

	All > 24 weeks PMA n = 279	Swiss n = 171	Europe n = 74	Non-European n = 34	χ^2^ or F	*p*-value	*p*-value corrected	Cramér’s V or ε^2^
**Rate of follow-up,** **n(%)**	279(92)	171(95)	74(89)	34(90)	3.53	.149	0.592	0.108
**NDI at 18 months, n(%)**	67(24)	40(23)	20(27)	7(21)	0.628	.730	0.854	0.048
**Neurodevelopment -al therapy, n(%)**	127(46)	80(47)	32(43)	15(46)	0.31	.859	0.859	0.03
**BSID-II Mental (MDI), M(SD)**	91.9(14.1)	92.84(13)	89.27(16)	94.90(18)	0.87	.421	0.730	0.02
**BSID-II psychomotor (PDI), median(IQR)**	87(77-97)	87(78-95)	87(77-102)	88.5(71-102)	0.58	.747	0.854	0.01
**BSID-III cognitive, median(IQR)**	100(90-110)	100(95-115)	95(90-105)	90(85-100)	12.42	.002**	0.016*	0.07
**BSID-III language, median(IQR)**	91(79-100)	91(83-103)	91(79-94)	83(77-100)	3.01	.222	0.592	0.02
**BSID-III motor, median(IQR)**	94(85-100)	97(88-100)	94(80.5-100)	91(85-100)	1.57	.456	0.730	0.01

Note. * p < .05 **p < .01.

**Table 5:** Neurodevelopment according to parental SES.

	High SES n = 106	Middle SES n = 114	Low SES n = 53	χ2 or F	*p*-value	*p*-value corrected	Cramér’s V or ε2
**Rate of follow-up, n(%)**	106(95)	114(94)	53(91)	0.76	.700	0.778	0.05
**Neurodevelopmental impairment at 18 months, n(%)**	23(22)	29(26)	15(29)	1.05	.592	0.778	0.06
**Neurodevelopmental therapy, n(%)**	47(44)	55(48)	23(44)	0.506	.778	0.778	0.04
**BSID-II Mental (MDI), median(IQR)**	97(84.5-104)	90.5(81-99)	92(82-98)	2.78	.249	0.664	0.03
**BSID-II psychomotor (PDI), median(IQR)**	87(80.5-101)	87(77.3-95)	86(70.5-101)	1.31	.519	0.778	0.01
**BSID-III cognitive, Mdn(IQR)**	100(86.3-105)	100(95-110)	95(85-100)	6.97	.031*	0.248	0.04
**BSID-III language, median(IQR)**	91(77.5-103)	91(83-100)	91(79-97)	0.92	.633	0.778	0.01
**BSID-III motor, median(IQR)**	94(82.8-100)	97(88-103)	91(82-100)	2.97	.227	0.664	0.02

Note. *p <.05.

**Table 6:** Neurodevelopment according to parental language.

	Language = French n = 186	Language = other n = 89	χ^2^ or t	p-value	p-value corrected	Cramér’s V or Cohen’s d
**Rate of follow-up, n(%)**	186(95)	89(91)	1.8	.210	0.279	0.78
**Neurodevelopmental impairment at 18 months,** **n(%)**	40(22)	27(31)	2.64	.132	0.264	0.10
**Neurodevelopmental therapy, n(%)**	80(43)	46(52)	1.96	.194	0.279	0.08
**BSID-II mental (MDI), mean(SD)**	93.10(15.6)	89.62(13.1)	1.37	.244	0.279	0.02
**BSID-II psychomotor (PDI), median(IQR)**	87(78.3-95)	86(71-102)	0.02	.876	0.876	0.0002
**BSID-III cognitive, median(IQR)**	100(90-111)	95(85-100)	6.92	.009**	0.036*	0.04
**BSID-III language, median(IQR)**	91(83-103)	86(77-94)	9.37	.002**	0.016*	0.05
**BSID-III motor, median(IQR)**	97(88-100)	91(79-100)	3.64	.056	0.149	0.02

Note. **p < .01.

## Discussion

This Swiss population-based study examined the association of SDoH with the short- and long-term outcomes of EPT infants. Overall, the findings show that maternal nationality, parental SES and language were mostly not associated with the perinatal management and short-term outcomes in neonatology but were associated with neurodevelopment at 18 months.

### SDoH and perinatal management

First, there was no identified differences in antenatal care (i.e., prenatal steroids, cesarean delivery, inborn status) or discharge destination (i.e., transfer to another hospital or home) based on maternal nationality, parental SES or language. Unlike findings from other contexts [33], no variations in management strategies were observed.

The main difference between groups was the significantly higher rate of multiple pregnancies among high SES and Swiss patients, consistent with findings from other cohorts [11]. Specifically, multiple pregnancies were more common among Swiss patients (34%) compared to European (27%) and non-European patients (14%). Similarly, the rate varied by SES, with 45% in the high SES category, 28% in the middle SES category, and 19% in the low SES category. We hypothesized that a proportion of these multiplet infants were conceived through in vitro fertilization (IVF), given that 7.1% of IVF pregnancies in Switzerland in 2022 resulted in multiplets [34]. Since IVF costs are most often not covered by the health insurance in Switzerland [35], individuals from lower socioeconomic backgrounds may face greater barriers to access these services.

### SDoH and short-term outcomes

There was no association of maternal nationality, parental SES and language, with most of the studied neonatal short-term outcomes, including mortality, necrotizing enterocolitis, sepsis, ROP, and severe brain lesions. Regarding mortality, while Wanner et al. [16] identified an association between nationality, socioeconomic group, and neonatal mortality in a Swiss national population-based study, there was no such association in our population.

Nevertheless, patients from the middle SES group were more likely to need oxygen at 36 weeks PMA compared to those in the high- or low-SES groups, without a clear explanation for this observation. Lower rates of BPD have been described in SDoH disadvantaged patients by Brumbaugh [36], who argues that treatment-based diagnoses may be biased by clinical decision-making rather than true physiological differences.

In addition, as described in other populations [13,37–39], the rate of feeding with MOM at discharge was significantly related to SES, with infants from high SES receiving MOM at discharge nearly twice as often as those from the lowest SES group. Most of the publications reporting similar findings stem from the USA, where ethnicity, short maternity leave and insurance status play an important role [40]. Despite universal maternity leave in Switzerland, there were differences in MOM provision, possibly linked to unmeasured social determinants.

This finding is particularly relevant because inflammation is increasingly recognized as a key contributor to BPD pathogenesis [6] and MOM has anti-inflammatory and immunomodulatory properties that may mitigate this risk. Thus, SES-related differences in MOM provision could contribute, at least in part, to the observed variation in BPD rates across socioeconomic groups. Additionally, both BPD and suboptimal MOM exposure are, in turn, linked to poorer neurodevelopmental outcomes [41,42], suggesting that SES-related disparities in neonatal care may have consequences that extend well beyond the immediate postnatal period.

Overall, these findings suggest that in our cohort, SES seemed to be the main driver of health inequality in the neonatal period, consistent with the results described by Racape et al. [43]. They also point to minimal differences in care management across the different groups, emphasizing the uniformity of treatment regardless of the nationality, SES or language of the EPT infant that is cared for in the neonatal intensive care unit. They also highlight the overall quality of the healthcare system in Switzerland, including its perinatal accessibility and insurance coverage.

### SDoH and neurodevelopment

Patients were examined at a mean corrected age of 19.5 months with a high follow-up rate of 92.5%. Contrary to what has been described in other populations [44], the rate of follow-up was similar across the measured SDoH.

There was no association of maternal nationality, parental SES or language, with the rate of a composite outcome of NDI, defined by either a BSID-II mental index <70, a BSID-III cognitive composite score <80, a BSID-III motor composite score <78 [26], or a BSID-III language composite score < 85, a severe visual impairment, or a hearing loss or CP grade >1. Nevertheless, there was an association between maternal nationality, parental SES and language and specific BSID-III sub-scores. Swiss patients had a significantly higher median BSID-III cognitive score compared to non-European patients. Likewise, patients from the middle SES group had a higher median cognitive score than those from the low SES group. Additionally, French-speaking children had significantly higher median BSID-III cognitive and language scores compared to non-French-speaking ones, aligning with findings from studies in Canada or France [45,46].

Mechanistic pathways proposed in the literature by which social adversity may be linked to neurodevelopment include systemic inflammation and epigenetic programming [47], antenatal nutrition [48], or poor maternal mental health [49], all of which may influence brain development and contribute to socioeconomic gradients in cognitive and language outcomes. While we did not directly evaluate these pathways, their recognition provides important context for interpreting the associations observed in our population.

### Strengths and limitations

A key strength of our study is the comprehensive population-based data collection, with a high follow-up rate, allowing for robust analysis of multiple variables. However, limitations include the potential for unmeasured confounding factors. Additionally, while we categorized nationalities and languages into broad groups, there may be intra-group variations that were not captured. Some of our categories included fewer patients than others, introducing potential bias into the analyses. The relatively small population size made it challenging to compare each SDoH with complete accuracy. Additionally, several SDoH were not available for analysis, such as neighborhood or ethnicity, which may be associated with outcome through different mechanisms, such as racism or hospitalization in lower care hospitals [50].

## Conclusion

In conclusion, while our study highlights the complex interplay between maternal nationality, parental SES and language in determining the health and neurodevelopmental outcomes of EPT infants, it also suggests that these factors may not unanimously lead to significantly worse outcomes. The overall findings point toward equity in neonatal care delivery across social groups, yet disparities remain in areas such as MOM provision and neurodevelopmental outcomes. Further research is needed addressing targeted interventions and policy changes to reduce the impact of social disadvantage and optimize both short- and long-term health outcomes in this vulnerable population [51–53]. This study provides a foundation for future work aimed at understanding and addressing the nuances of these important SDoH.

## Abbreviations

BSID-II, BSID-III: Bayley Scales of Infant Development, 2nd and 3rd edition
CP: cerebral palsy
EPT: extremely preterm
GA: gestational age
HUS: head ultrasound
IVF: in vitro fertilization
MOM: mother’s own milk
NDI: neurodevelopmental impairment
PMA: post-menstrual age
ROP: retinopathy of prematurity
SDoH: social determinants of health
SES: socioeconomic status

## Supporting information

S1 Data SDOH_EPT_codes. (XLSX)

S2 Data SDOH_EPT_details. (XLSX)
